# Multiple Recurrent Infective Endocarditis Secondary to Streptococcus mitis Bacteremia Despite Proper Antibiotic and Surgical Treatment

**DOI:** 10.7759/cureus.38981

**Published:** 2023-05-13

**Authors:** Zhongying Liu-An, Vladimir Joseph, Stacey Damito, George Stoupakis

**Affiliations:** 1 Medicine, Hackensack University Medical Center, Hackensack, USA; 2 Cardiology, Hackensack University Medical Center, Hackensack, USA

**Keywords:** aortic valve abscess, redo surgical aortic valve replacement, surgical aortic valve replacement (savr), recurrent infective endocarditis, s mitis endocarditis

## Abstract

Infective endocarditis (IE) is a rare and potentially fatal disease. It is an infection of the endocardium of the heart and heart valves. One of the major complications faced by patients who have recovered from a first episode of IE is recurrent IE. Risk factors for recurrent IE include intravenous (IV) drug use, prior episodes of IE, poor dentition, recent dental procedures, male gender, age over 65, prosthetic heart valve endocarditis, chronic dialysis, positive valve culture(s) obtained at the time of surgical intervention, and persistent postoperative fever. We present a case of a 40-year-old male with a history of former IV heroin use who experienced multiple episodes of recurrent IE caused by the same pathogen, Streptococcus mitis. This recurrence occurred despite the patient completing the appropriate course of antibiotic therapy, undergoing valvular replacement, and maintaining drug abstinence for two years. This case highlights the challenges associated with identifying the source of infection and emphasizes the need to develop guidelines for surveillance and prophylaxis against recurrent IE.

## Introduction

Infective endocarditis (IE) is a rare and potentially fatal disease with an annual incidence of about 3 to 10 per 10,000 people and an in-hospital mortality rate of around 20% [1]. It is an infection of the endocardium of the heart, often involving cardiac valves, most commonly the mitral valve. IE is frequently precipitated by bacteremia, which crosses the endocardium and triggers inflammation and subsequent damage. Risk factors for IE include older age, poor dental hygiene, prior history of IE, intravenous (IV) drug use, structural heart or valvular disease, congenital heart disease, indwelling catheters, and cardiac implant devices. The most common causative agent of IE is Staphylococcus aureus, accounting for 30% of cases, followed by Viridans Group Streptococci, which account for approximately 20% of cases [2]. Streptococcus mitis is a species of Viridans Group Streptococci and a normal colonizing agent of the human oropharynx. It has the highest prevalence of IE among Viridans Group Streptococci [3]. Patients with IE typically present with symptoms such as fever, chills, night sweats, fatigue, chest pain, and myalgias. To aid in diagnosis, clinicians use the Duke Criteria as a tool to assess the likelihood of IE [4].

When there is a high suspicion of IE, management involves initiating IV antibiotics as soon as blood cultures are drawn. Treatment of endocarditis on native valves usually requires two to six weeks of IV antibiotic therapy, while prosthetic valve endocarditis typically necessitates six weeks of IV antibiotic treatment. Surgery to remove the nidus of infection or replace a damaged valve is considered when patients have severe complications of IE, such as congestive heart failure, severe aortic or mitral valve regurgitation, periannular extension, systemic emboli, or persistent sepsis [5]. Recurrent IE is a serious complication of IE with a high mortality rate. Despite proper treatment, approximately 5% to 10% of patients still develop recurrent IE [6,7].

## Case presentation

A 40-year-old male presented to the ED with two weeks of fever, chills, and night sweats. He had a history of recurrent Streptococcus mitis IE, requiring aortic valve (AV) replacement (AVR) and re-do AVR, abscesses of the aortic root and mitral annulus, and former IV heroin use.

The patient was first diagnosed with Streptococcus mitis IE and peri-aortic valvular abscess in March 2018 while actively using IV heroin. At that time, he underwent biologic AVR with irrigation and plication of the AV abscess, leading to eventual resolution. In August 2020, he experienced a nearly identical recurrence, with multiple vegetations found on both the prosthetic aortic and native mitral valves. He underwent multiple interventions, including re-do AVR (27 mm Inspiris), primary repair of the aortic root and mitral annular abscesses, and mitral valve vegectomy. In both instances, the patient received six weeks of penicillin and ceftriaxone treatment based on antibiotic susceptibility panels (Table 1) and following recommendations for IE management.

**Table 1:** Antibiotic susceptibility testing. Streptococcus mitis was sensitive to penicillin and ceftriaxone both in March 2018 and August 2020. MIC: Minimum inhibitory concentration

Date	Antibiotics	Streptococcus mitis group MIC (μg/mL)	Sensitivity
March 2018	Ceftriaxone	0.064	Sensitive
	Erythromycin	0.094	Sensitive
	Penicillin	0.032	Sensitive
	Vancomycin	0.75	Sensitive
August 2020	Ceftriaxone	0.032	Sensitive
	Erythromycin	4	Resistant
	Penicillin	0.023	Sensitive
	Vancomycin	1	Sensitive

**Figure 1:**
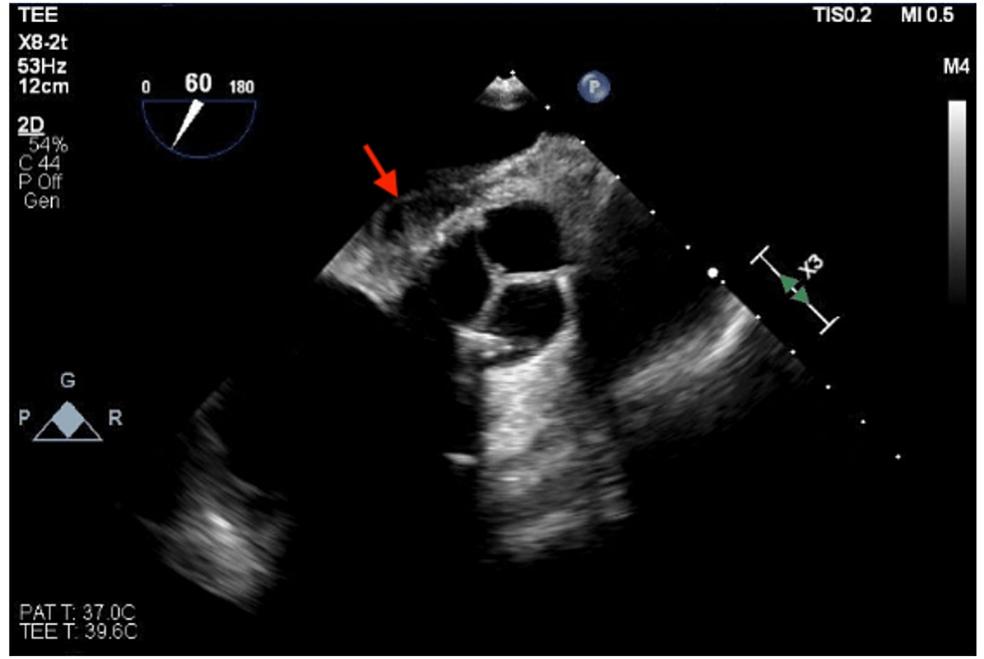
Transthoracic echocardiogram. Transthoracic echocardiogram showed a perivalvular echolucent area between the aortic valve and left atrium concerning an aortic root abscess (red arrow).

**Figure 2:**
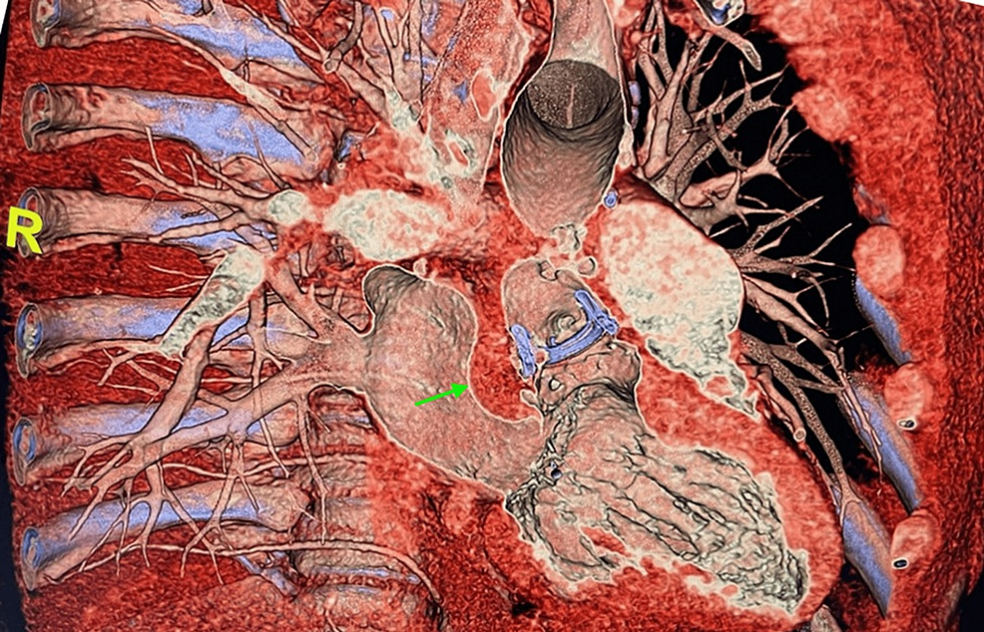
Cardiac computed tomography. Cardiac computed tomography showed a hypoattenuating tissue measuring 3.1 x 2 cm in the potential space between the aortic root at the level of the noncoronary leaflet and the anterior wall of the left atrium which may represent a perivalvular abscess (green arrow).

**Figure 3:**
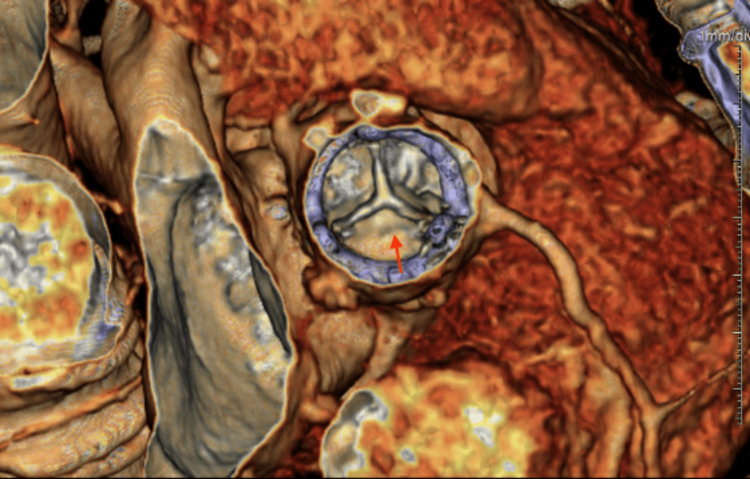
Cardiac computed tomography. Cardiac computed tomography showed a 0.6 cm hypoattenuating leaflet thickening along the noncoronary cusp of the prosthetic AV (red arrow), likely due to perivalvular thrombus or pannus. AV: Aortic valve

On this admission, the patient denied any IV drug use or recent dental procedures. He had a fever of 100.6°F. The physical exam was only noticeable for a 1/6 systolic murmur at the left sternal border, with no skin rash noted. Initial labs showed a normal white blood cell count, an elevated sedimentation rate (74 mm/h, reference: 0-15), and an elevated C-reactive protein (18.6 mg/dl, reference: <0.5). The respiratory pathogen panel and urinalysis were negative for infection. The urine drug screen was only positive for cannabinoids. However, his blood culture again showed positive growth for Streptococcus mitis (Table 2). A repeat transesophageal echocardiogram revealed a preserved left ventricular ejection fraction of 40-45% and a periaortic abscess between the aortic root and left atrium, without evidence of valvular vegetation (Figure 1). Cardiac computed tomography (CT) revealed a 3.1 x 2 cm periaortic abscess between the aortic root and the anterior wall of the left atrium (Figure 2), as well as a 0.6 cm hypoattenuating leaflet thickening (HALT) along the noncoronary cusp of the prosthetic AV, likely due to perivalvular thrombus or pannus (Figure 3). Nevertheless, an infectious disease specialist was consulted, and the patient was started on IV ceftriaxone, vancomycin, and rifampin for empiric treatment of IE.

**Table 2:** Antibiotic susceptibility testing in June 2022. Streptococcus mitis was sensitive to penicillin and ceftriaxone. MIC: Minimum inhibitory concentration

Date	Antibiotics	Streptococcus mitis group MIC (μg/mL)	Sensitivity
June 2022	Ceftriaxone	0.064	Sensitive
	Erythromycin	1.5	Resistant
	Penicillin	0.032	Sensitive
	Vancomycin	0.5	Sensitive

Given that the patient had abstained from IV drug use since August 2020, the etiology of his bacteremia needed clarification. Unfortunately, a thorough infectious workup was unrevealing. Dentistry was consulted, and a formal dental exam did not identify any active periodontal disease or tooth abscess. However, the patient appeared to maintain poor oral hygiene. Oral maxillofacial surgery was also consulted, and computed tomography of the facial bones was negative for osteomyelitis or dental lesions.

The rest of his hospital course was uneventful. He remained afebrile, and repeat blood cultures were negative for growth. The patient was discharged on six weeks of ceftriaxone and two weeks of gentamicin based on antibiotic sensitivities, with weekly outpatient lab follow-up and eventual overall recovery.

## Discussion

Recurrent IE is a feared complication of IE. When recurrence is caused by the same species within six months of the initial episode, it is considered a relapse. When it is caused by the same or different species after six months from the initial episode, it is considered a re-infection [8]. Our patient likely had a re-infection with the same pathogen, Streptococcus mitis, the most common cause of IE among VGS [9]. In patients with native heart valves and IE caused by a penicillin-susceptible VGS strain, it is reasonable to treat with four weeks of IV penicillin or ceftriaxone. When hosting penicillin-resistant VGS, two weeks of gentamicin are usually added for synergistic benefit. Similarly, patients with prosthetic valves require six weeks of IV penicillin or ceftriaxone, with or without gentamicin in the first two weeks [4]. Our patient received complete courses of IV antibiotics at every encounter, valvular replacement with repair, abscess treatment, and IV drug abstinence per a negative toxicology. However, he inexplicably developed recurrent bacteremia and IE with the same pathogen. Although it is not uncommon for patients to have multiple episodes of recurrent IE, it is atypical for Streptococcus mitis to reappear after two years without some dormant nidus of infection [10,11]. This unusual presentation prompted us to further explore other underlying causes that might have contributed to his recurrent IE. 

Risk factors of recurrent IE have been identified to be associated with IV drug use, prior episodes of IE, poor dentition, recent dental procedures, male gender, older age greater than 65, prosthetic heart valve endocarditis, chronic dialysis, positive valve culture(s) obtained at time of surgical intervention, and persistent postoperative fever [12]. Patients with more than two risk factors are more likely to suffer from recurrent IE than those who have only one risk factor [13]. Our patient presented with multiple risk factors. Streptococcus mitis is the most common inhabitant of the oral cavity, including dental plaque and the upper respiratory tract. Although a formal dental evaluation performed at presentation did not identify any active periodontal disease or tooth abscess, the patient's poor oral hygiene could still serve as a reservoir of bacteria. Even if the patient denied any recent dental procedure or trauma to his oral cavity, bacteria could still have entered the bloodstream through bleeding caused by routine tooth brushing and flossing. The patient's cardiac CT revealed a 0.6 cm HALT along the noncoronary cusp of the prosthetic AV, which was likely due to perivalvular thrombus or pannus. Bacteria could adhere to the thrombus or pannus using their surface protein, adhesin, and form a nidus of infection [14]. When bacteria colonize the endocardial tissue, they form a biofilm consisting of bacterial aggregates in a self-produced extracellular polymeric matrix [15]. The deep-seated biofilm can cause limited antibiotic penetration into the bacterial vegetation in IE [16]. This might partially explain why the patient still developed recurrent IE despite proper antibiotic treatment. 

The patient underwent biologic AVR in March 2018 and a re-do AVR after multiple vegetations were found on the prosthetic valve in August 2020. He did not have any follow-up examinations, imaging studies, and laboratory tests to monitor the clearance of the infection, valvular function, and abscess and vegetation resolution until this admission. His multiple episodes of recurrent IE might suggest that the surgical treatment was not fully adequate. Long-term monitoring is crucial to evaluate the effectiveness of the surgical outcome. Lastly, IV drug use imposes a high risk of recurrent IE [17]. The patient did not follow up with outpatient addiction medicine after discharge. Although his urine toxicology at presentation was only positive for cannabinoids, it remains unknown if he was using other IV drugs that were not detected on the test.

## Conclusions

Our patient presented with a unique case of recurrent IE with the same pathogen, Streptococcus mitis, despite proper IV antibiotic treatment, valve replacement, valvular abscess intervention, and abstinence from IV drug use for two years. The source of the infection remains unclear after a thorough workup. This case serves as a reminder to clinicians that prevention of recurrent IE continues to be challenging, even with diagnostic advancements and adequate treatment. Future surveillance and prophylactic guidelines are needed to lower the risks of recurrent IE.
